# Self-organization in amoeboid motility

**DOI:** 10.3389/fcell.2022.1000071

**Published:** 2022-10-14

**Authors:** Andrew Callan-Jones

**Affiliations:** Laboratoire Matière et Systèmes Complexes, Université de Paris Cité, Paris, France

**Keywords:** actomyosin cortex, cortical flow, surface mechanics, active gel theory, mechanochemical feedback

## Abstract

Amoeboid motility has come to refer to a spectrum of cell migration modes enabling a cell to move in the absence of strong, specific adhesion. To do so, cells have evolved a range of motile surface movements whose physical principles are now coming into view. In response to external cues, many cells—and some single-celled-organisms—have the capacity to turn off their default migration mode. and switch to an amoeboid mode. This implies a restructuring of the migration machinery at the cell scale and suggests a close link between cell polarization and migration mediated by self-organizing mechanisms. Here, I review recent theoretical models with the aim of providing an integrative, physical picture of amoeboid migration.

## 1 Introduction

Long before the molecular components of cell migration were known, Abercrombie et al. (1970) observed signs of large-scale motion at the cell surface, and speculated that generic surface movements, in the presence of a substrate, would propel the cell forward.

Since then, experimental studies of solid substrate-based cell crawling, known as mesenchymal migration, have revealed key molecular players and processes: F-actin polymerization at the cell leading edge, formation of specialized adhesion clusters, and actomyosin contractility at the cell rear to enable locomotion Blanchoin et al. (2014). These studies have inspired theoretical models with broad relevance, including reaction-diffusion patterns and waves associated with actin polymerization kinetics Allard and Mogilner (2013); the Brownian ratchet model of cell leading edge motion Peskin et al. (1993); and the molecular clutch model of retrograde actin flow engagement with focal adhesions Chan and Odde (2008).

Yet, in recent years *in vivo* and *in vitro* studies have identified a set of migration modes—grouped together as “amoeboid migration” Paluch et al. (2016)— that demand a re-thinking of the physical principles of cell motility. Somewhat ironically, these new modes, which involve cell-scale surface flows and deformations, invite a reappraisal of generic mechanisms hinted at in early migration studies Abercrombie et al. (1970). While all types of cell migration require some kind of rearward directed element at the cell surface mechanically communicating with a substrate, amoeboid migration is usually characterized by the absence of specialized adhesion complexes. To move in a variety of *in vivo* settings where adhesion complexes are not feasible, the cell surface must use different means of momentum transfer with its surroundings. Luckily, various cell types have evolved different modes of surface movement, either in the plane or out of the plane, and adapted to a particular environment, in order to propel themselves. While these different surface behaviors have been documented, the large-scale physical mechanisms that organize these behaviors and by which propulsion force is generated are not widely known.

The first goal of this paper is to shine light on recent theoretical advances on how cell surface movements enable migration. I start by discussing what one might call feed-forward models, which reply to the question, given a specified internal motile activity (whose existence is assumed), what are the emergent behaviors imposed by the constraints of physical laws that give rise to motion? Examples of these models are presented in Sections 2 and 3. There, I highlight the interactions between cell surface and environment geometry in influencing the migration mode. The second goal is to provide some insight into the self-organized behaviors that underlie amoeboid migration. The innate capacity of a cell to switch its migration mode implies that, in response to some cue, interactions between surface components change to cause large-scale changes in how the cell polarizes and moves. Therefore, feedback mechanisms that drive cell polarization are closely related to migration Goehring and Grill (2013); Hannezo and Heisenberg (2019). In section 4, I review an example of this behavior for a one-dimensional cortex, which showcases the dual role of actin flows in patterning the cell cortex Callan-Jones and Voituriez (2016); Illukkumbura et al. (2020) and in driving migration. Extrapolating from this example to a fluid tubular surface, I sketch how coupling mechanochemical feedback with surface deformation can generate peristaltic wave-based migration, a mode that, to my knowledge, has hardly been explored Franz et al. (2018).

The surface mechanics involved in amoeboid migration rely on an active component—such as an actomyosin cortex—coupled to a plasma membrane. There is mounting evidence for possible roles played by the membrane in motility Lieber et al. (2013); O’Neill et al. (2018); Aoun et al. (2020). However, the physical understanding of how membrane dynamics and tension are integrated into the migration machinery is incomplete. In the model examples below, the cell surface is treated as a composite structure, and the plasma membrane is not specifically considered. Its impact on migration will be left for future work.

Some of the references I discuss below have been reviewed elsewhere [see for example, Paluch et al. (2016); Bodor et al. (2020)], but not, as far I can tell, from the perspective of theoretical modeling. Also, some have appeared in physics journals, and therefore might not be well-known to a wider community working on cell migration. With the benefit of hindsight, I have attempted to identify unifying ideas that connect different modeling approaches that have appeared in the past decade.

## 2 Actin flow-based models in confined settings

*In vitro* studies of different types of confined cells have revealed a simple yet versatile mode whereby the cell maintains a fixed shape and its migration is driven by cell-scale cortical flows directed from the cell front to the rear Liu et al. (2015); Ruprecht et al. (2015); Bergert et al. (2015). The propulsion force has been attributed to non-specific adhesion Paluch et al. (2016), which can be modeled as a friction force Bergert et al. (2015). Since the mechanism here depends on flowing actomyosin that transiently binds to a flat surface, it has some points in common with models of mesenchymal crawling Kruse et al. (2006); Rubinstein et al. (2009). The theory of Bergert et al. (2015) is therefore a useful starting point to discuss amoeboid models.

### 2.1 Migration in quasi-one dimensional straight channels

Microchannels provide a controlled means to study the effect of confinement on cell migration. Heuzé et al. (2011). These systems are also useful because the cell shape is fixed and the actin flows are essentially one-dimensional, making the modeling simpler and the theoretical concepts easier to illustrate. A handful of quasi-one dimensional models have been formulated that predict relationships between actin flow and cell motion Callan-Jones and Voituriez (2013); Recho et al. (2013); Kimpton et al. (2013). The basic approach of these models is to consider actomyosin as an active fluid Jülicher et al. (2007); Marchetti et al. (2013); Prost et al. (2015); Jülicher et al. (2018) and write down differential equations expressing mass conservation and force balance. However, these coupled equations can be difficult to solve (see Sec. 4), and can obscure the physics of amoeboid migration in channels. An elegant approach was taken in Bergert et al. (2015) that bypasses the interdependencies of cortical actin flow and concentration fields, and assumes a given myosin-related active stress profile as an input that drives flow. I will review, and attempt to simplify, the model in Bergert et al. (2015), as the physical concepts it exposes are general and form a basis for more complex types amoeboid migration.

**FIGURE 1:**
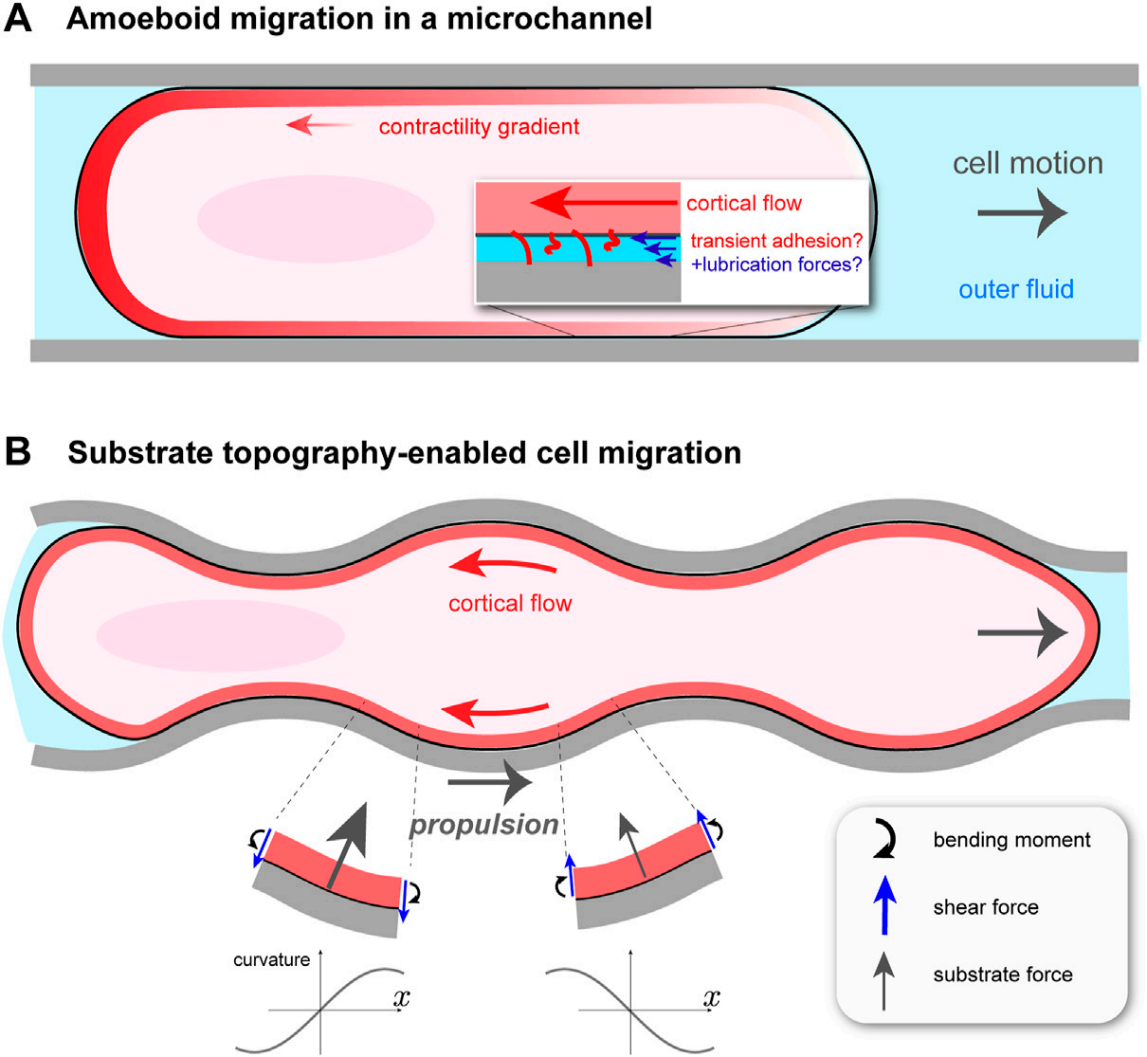
Cortical flow-based migration in confinement. **(A)** Illustration of non-specific friction mediated cell motion in a cylindrical microchannel Bergert et al. (2015). Rearward cortical flow is driven by a gradient in contractility *ζ*(*x*), and momentum is thought to be transferred to the surroundings by transient adhesion and lubrication forces. The channel is filled with fluid, which hydraulically resists cell migration. **(B)** Migration of talin knock-out leukocyte in a wavy channel Reversat et al. (2020). Substrate topography forces the cortical flow to be curvilinear, creating bending stresses and, as a result, an asymmetric profile in normally-oriented substrate forces. This gives rise to a net propulsion force in the direction opposite to that of the cortical flow.

Confined Walker carcinoma cells were observed to undergo cortical flow-driven motion in long, cylindrical channels filled with fluid Bergert et al. (2015). This is distinct from previous microchannel migration studies, for example, actin-based “chimneying” in dendritic cells Hawkins et al. (2009). Channels were coated with different proteins to explore the influence of non-specific friction on migration. The cell was modeled as a self-propelled sphero-cylinder with constant shape and constant speed *U*, as shown in Figure 1A. It is assumed to make contact with the channel walls over a cylindrical segment of length *L* and radius *R*. The contact-free front and rear of the cell are treated as half-spheres of radius *R*. Assuming axisymmetry, the cortical flow on the contact region is one-dimensional between *x* = 0 (rear) and *x* = *L* (front), and is obtained from a local balance of forces in the cortex. There are three: viscous forces due to internal dissipation; transient, non-specific adhesion between the cortex and the channel walls, modeled as a friction force; and active force due to myosin contractility. This balance is expressed as *ηd* ^2^ *v*/*dx* ^2^ − *α*(*v* + *U*) + *dζ*/*dx* = 0. Here, *v*(*x*) is the cortical velocity in the cellular reference frame moving at speed *U*; *η* is the cortical viscosity and *α* is a friction coefficient, which is assumed constant over the contact region. The active force is equal to the gradient in active stress *dζ*(*x*)/*dx* < 0, which is what drives flow from front to rear. The main purpose of the model is to relate *U* to measurable parameters such as *α* and the active stress *ζ*(*x*), taken to be proportional to cortical myosin II concentration. Solving the differential equation for *v*(*x*) requires knowledge of two unknown constants, in addition to *U*. These are determined by the requirement that the total external force (again neglecting inertia) on the migrating cell is zero and by matching the cortex stresses at the edges of the contact region with the end caps. Total force balance is $\pi R^{2}P_{\text{cell}}-2\pi R\int_{0}^{L}\alpha \left(v+U\right)\;dx=0$ , where *P*_cell_ is the difference in fluid pressure on the cell between the rear and the front. If the pressures are the same at the entrance and exit of the microchannel then the pressure difference is solely due to autonomous cell motion and *P*_cell_ = −*α*_*D*_ *U*. Here, *α*_*D*_ is a drag coefficient related to the relative motion between the cell and the outer fluid. The possible sources of this drag include cell membrane permeability Stroka et al. (2014); Li et al. (2020), induction of velocity gradients in the fluid wedged between the channel walls and the cell front and rear, as well as hydraulic resistance of fluid farther away being pushed over the channel walls.

In this model, the speed *U* is directly related to the contractility gradient: *U* ∝Δ*ζ* ≡ *ζ*(0) − *ζ*(*L*). Bergert et al. (2015) show this for the simple case of a linear function for *ζ*(*x*), but it can be generalized to any monotonically decreasing *ζ*(*x*) using Green’s functions Mathews and Walker (1970). Even in the linear case, the general dependence of *U* on the various parameters in the problem is involved, but a clear picture is obtained in two limiting situations. If the non-specific friction *α* is weak, then *U* ∼Δ*ζαL* ^2^/(*ηα*_*D*_ *R*). This shows that, for a given cell volume, by increasing the contact region *L*, confinement can aid migration even if cell traction is weak. In the opposite limit, of large friction and therefore negligible actin flow (in the lab frame), *U* ∼ *R*Δ*ζ*/(*η* + *α*_*D*_ *R* ^2^). In this case, cell displacement occurs by a continuous blebbing-like process whereby the less contractile cell front is expanded while simultaneously the more contractile cell rear is contracted.

It might seem surprising that *U* does not depend explicitly on actin turnover. This is because there is no cortical actin density dependence in this model: contractility is specified *a priori* and there is no in-plane pressure term. The neglect of pressure follows from the assumption that the cortex is perfectly compressible in the plane; see, also, Recho et al. (2013). This can be justified because on long enough timescales fast turnover within the cortex maintains a roughly constant two-dimensional density, independent of in-plane flow Jülicher et al. (2018). Of course, *U* depends implicitly on turnover, otherwise no moving steady-state would exist.

A useful readout illustrating how forces enable migration is obtained by looking at the spatial distribution of traction stresses the cell exerts on its surroundings. This can also be interesting as a way to frame amoeboid migration more generally in the context of self-propelled systems, such as bacteria and synthetic systems Bechinger et al. (2016). Self-propelled particles can be distinguished by the way they interact with their surroundings, and can be “pushers” or “pullers”. One can determine to which class a migrating cell belongs from the sign of the dipole moment, *Q*, of the forces the cell exerts. Quantitatively, in Bergert et al. (2015), this is given by $Q=2\pi R\int_{0}^{L}x\;\left[f\left(x\right)-\left\langle f\right\rangle \right]\;dx$ , where *f*(*x*) = *α*(*v*(*x*) + *U*) is the traction stress (force per unit area) the cell exerts on the channel walls and ⟨*f*⟩ is its average over the contact region. It is readily seen that $Q=2\pi R\;\alpha \int_{-L/2}^{L/2}x\;v\left(x\right)\;dx$ , and thus the sign of *Q* is related to the asymmetry of cortical flow with respect to the middle of cell. One can show that the asymmetry is related to the curvature *d* ^2^ *ζ*/*dx* ^2^, and that *Q* > 0 if *ζ*(*x*) is a monotonically decreasing, concave-up function of *x*, a point not made explicitly in Bergert et al. (2015). Based on their measurements of myosin II concentration, this condition appears to hold, and, therefore not coincidentally, Bergert et al. (2015) measure *Q* to be positive, and increases with surface adhesion. Confined, migrating Walker carcinoma cells are therefore pushers, reflecting the strong localization of myosin activity near the cell rear enabling the cell to push back on its environment. This is in direct contrast with mesenchymally crawling cells, such as keratocytes, which behave as pullers, with *Q* < 0 Kruse et al. (2006); Schwarz and Safran (2013). For crawling cells, the motile machinery is localized at the lamellipodial leading edge, allowing the cells to pull themselves forward. Thus, the dipole moment provides a simple metric of the force distribution migrating cells exert and is revealing of the internal machinery of force generation.

An interesting question is whether there is, in practice, a minimum adhesion necessary for channel-based amoeboid migration. By coating the channel walls with different treatments so as to vary *α*, Bergert et al. (2015) found that the maximum traction stresses obtained were in the range of Pascals, orders of magnitude lower than in mesenchymal crawling Paluch et al. (2016). Moreover, in the most slippery of channels, with tractions in the milli-Pascal range, cells did not move at all. This suggests that, even if the cell is strongly confined and with a significant contact length *L*, migration can be impeded if traction is not sufficient.

### 2.2 Topography-enabled amoeboid migration

A perfectly cylindrical microchannel does not fully capture the irregular, confined environment that cells encounter *in vivo*. One could then ask if, in addition to transient adhesion, other force-generating mechanisms exist for confined, migrating cells. It turns out that cells, such as those of the immune system, can exploit the varying topography of their surroundings to move Tozluoğlu et al. (2013); Paluch et al. (2016), circumventing the problem of insufficient adhesion. Yet, to what extent this type of migration uses cortical flow, as in microchannels, or requires environment-adapted actomyosin processes remained unclear. This problem was taken up in a recent paper by Reversat et al. (2020), who studied leukocyte migration in straight and wavy channels. The sides of the channels were curved, while the top and bottom were flat. By knocking out talin, a key component of integrin-based adhesion, they were able to create essentially zero adhesion conditions. Perhaps not surprisingly, by comparing wild type (WT) and talin knock-out (TKO) leukocytes, Reversat et al. (2020) found that TKO cells were immobile in straight channels. More interestingly, both cell types moved, and with about the same speed, in wavy channels, and the TKO cell speed increased with decreasing channel wavelength. In both cell conditions, cortical actin was found to undergo steady retrograde flow, and that in wavy channels this flow follows the channel topography. This observation suggests the tantalizing idea that the actomyosin cortex has an adaptive quality such that, in the absence of traction forces, its flows can harness the substrate shape in order to move.

How can cortical flow transmit momentum to a frictionless substrate to propel the cell forward? Everyday experience tells us that the bumps on anti-slip flooring convert normally-directed pushing into forward walking. In the case of fluid flowing tangentially to a wavy surface, one might be tempted to think that propulsion arises because the fluid is forced to bend and follow the substrate, and hence accelerates vectorially, resulting in a normal reaction force on the cell. This would be true at Reynolds’ numbers where inertia is not negligible, which is not the case here. The origin of the propulsion force is more subtle. A straightforward, but slightly laborious, way of finding the dependence of the propulsion force, *F*_prop_, on channel shape can be done by modeling the cortex as an undulating fluid layer and solving the Stokes equations of low Reynolds’ number hydrodynamics. Given an average retrograde cortical velocity of − *v*_0_ < 0 along the channel (*x*) axis and a vanishing shear stress—i.e., zero friction—at the cortex/substrate interface, this can be readily done Reversat et al. (2020). A more intuitive way, which gives a similar dependence on channel wavelength, is to regard the cortex as a very viscous, thin sheet. With this approach the underlying physics can be revealed without an assumption of sinusoidally-shaped channels, as is done in Reversat et al. (2020). I will outline here this alternative approach.

In considering the motion of the wavy cortex, it is useful to follow a particular fluid element. As it flows backward to a region with, say, increasing curvature *C*—that is, *dC*/*dt* > 0 in its reference frame and *dC*/*dx* < 0 in the lab frame—it feels an internal bending moment, *m*; see Figure 1B. At steady state, *m* = −*η*_*b*_ *v*_0_ *dC*/*dx*, where *η*_*b*_ > 0 is known as the *bending viscosity* Salbreux and Jülicher (2017). One can show that *η*_*b*_ ∼ *ηδ* ^3^ with *η* the cortical viscosity and *δ* the cortex thickness; *η*_*b*_ increases with cortex thickness, as expected intuitively. As in the elastic theory of solid plates Landau and Lifshitz (1986), balance of torques on a sheet element subject to an internal moment *m*(*x*) creates shear forces (per unit length perpendicular to the plane) *f* = −*dm*/*dx*. In turn, a balance of forces normal to the substrate requires a normally-directed force per unit area *P*(*x*) = −*df*/*dx* = −*η*_*b*_ *v*_0_ *d* ^3^ *C*/*dx* ^3^, on top of the constant value *P*_0_ present even if *v*_0_ = 0. Therefore, the main effect of coupling retrograde flow to an uneven substrate is to generate normal forces related to its topography. Assuming a sinusoidally modulated channel, one finds a force profile *P*(*x*) that is asymmetric between troughs and crests (Figure 1B). It is this asymmetry, related not to the channel shape but to the direction of retrograde flow, that generates a net force along *x*. For a wave shape with amplitude *ϵ* and wavelength *λ*, the curvature is *C*(*x*) = *ϵ*(2*π*/*λ*)^2^ cos(2*πx*/*λ*). Projecting the substrate forces along *x*, and averaging over one wavelength, one obtains the propulsion force *F*_prop_ = *η*_*b*_ *v*_0_ *ϵ* ^2^(2*π*/*λ*)^6^. It vanishes for a flat channel, i.e., *ϵ* = 0 or *λ* → *∞*, as it should since there is no friction force.

How does this propulsion force compare with previously documented values for amoeboid migration? We can make a quick estimate: taking *δ* = 200 nm Clark et al. (2013); *η* = 10^4^ Pa.s Bergert et al. (2015); *v*_0_ = 10 μm/min; *ϵ* = 1 μm; and *λ* = 10 μm, the topography-based propulsion force (per unit area) is on the order of Pascals. This is in the upper range for traction forces driving non-specific adhesion-based migration in smooth channels Paluch et al. (2016). This shows that, in the absence of adhesion, cells can use alternative strategies to generate sufficient force to migrate.

## 3 Using cell shape changes to move

A hallmark of amoeboid motility is the ability of cells to adapt their internal migration mechanism in response to an external cue. As a counterpoint to topography-based migration, where the environment shapes the cell, there is growing evidence that cells can also self-deform in order to move, either in straight channels (Section 3.1) or in a free fluid (Section 3.2). I consider here two types of model that illustrate how cell surface deformation can drive migration.

### 3.1 Shape-shifting motion in a confined space

**FIGURE 2:**
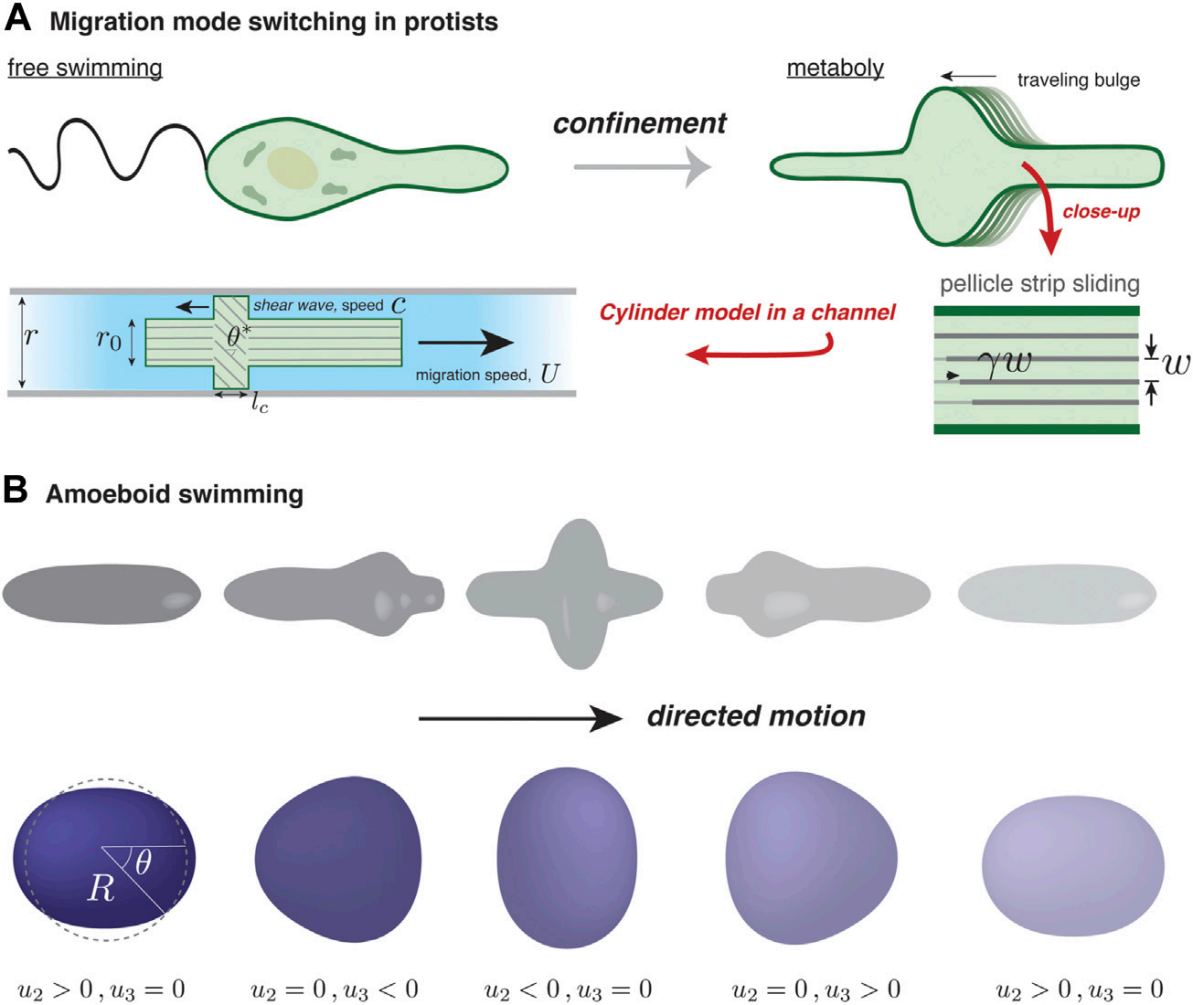
Self-deformation-based migration. **(A)** Depending on confinement, protists from the genus *Euglena* can swim using a flagellum or inch forward using surface waves, a process known as metaboly. The rearward traveling surface bulges characterizing metaboly are generated by microtubule activity in the parallel strips forming the pellicle, an organismic envelope. The strips are assumed to maintain a fixed separation, *w*, and internal activity generates a localized traveling pulse of relative strip sliding, *γ* Noselli et al. (2019). In the simple version of the model by Noselli et al. (2019), the confined organism is modeled as a uniform cylinder of radius *r*_0_, except over a region where *γ* is non-zero. There, and over a length *l*_*c*_, relative sliding induces tilting with respect to the channel axis and bulging of the surface to become in contact with the channel walls; the tilt angle is related to *r*_0_ and the channel radius by cos θ* = *r*_0_/*r*. In the frame of the moving cylinder, the contact zone moves rearward with speed *c*; the cell speed *U* depends solely on *c* and θ*. **(B)** Illustration of amoeboid swimming in a viscous fluid (not shown). Actively generated forces at the cell surface are assumed to give rise to cyclic cell shape changes. In order to respect Purcell’s scallop theorem Purcell (1977), directed motion requires that a forward-played movie of a “stroke” cycle looks different than its backward-played version. A minimal reduction of amoeboid swimming is a superposition of two deformation modes, one with nematic symmetry (*l* = 2) and another with polar symmetry (*l* = 3). The surface displacement away from the spherical state, *u*(*θ*, *t*), is projected onto the two modes, with amplitude *u*_2_(*t*) and *u*_3_(*t*).

It is known that Euglenids, single-celled protists, use different migration mechanisms depending on their environment. When *Euglena* are suspended in fluid they swim using their flagella, powered by microtubules; under confinement, they switch to a crawling mode that relies on regular pulses of surface deformation, known as metaboly Schaechter (2012); see Figure 2A. Other types of flagellates are also known to undergo a flagellate-to-amoeboid transition upon confinement Kusdian et al. (2013); Brunet et al. (2021). For *Euglena*, the amoeboid mode of migration involves active microtubule behavior, though the microscopic details are not fully known. A set of recent studies has sought to uncover the physics of metaboly-based migration Arroyo et al. (2012); Noselli et al. (2019).

Noselli et al. (2019) presented a multi-scale, experimental and theoretical study into how microtubule sliding on the cell surface, known as the pellicle, allows *Euglena* to move in narrow channels. They developed a model showing how shear strain of microtubules generates persistaltic-like surface pulses, which use the channel walls to propel forward. I will present the key ingredients and development of this model; it shows how generic concepts such as surface kinematics, conservation of surface area and of volume, when coupled to an intrinsic force generation mechanism, can drive cell motion.

In a simple version of their model of metaboly-based motion, Noselli et al. (2019) treat the organism as a cylinder of radius *r*_0_ confined in a channel of radius *r* ≳ *r*_0_. They represent the pellicle as an array of parallel strips separated by a fixed distance, *w*; see Figure 2A. A spatio-temporal pulse of relative sliding between adjacent strips, *γ*, travels at speed *c* in the cell’s reference frame from the prospective front to rear of the cell. Though the details are still not fully clear, this pulse is thought to be triggered by a controlled release/sequestration of subpellicular stores of calcium that activate microtubules. Since the strips are fixed in place at the front and rear poles of the cell, a shear displacement *γ* localized somewhere along the cell length is necessarily accompanied by a tilting of the strips by an angle *θ* ∝ *γ* with respect to the migration axis in that location. Furthermore, because the pellicle maintains a fixed strip spacing, the surface must deform outwardly where the strips are tilted. This is seen by noting that as the strips tilt, their spacing as measured along a contour perpendicular to the migration axis gets larger, and because the total number of parallel strips going around the cell remains the same, the local radius must increase. As a result, where the amplitude of *γ* is largest, the pellicle will expand so that it is in contact with the channel walls. On the contact region, a bit of geometry shows that the tilt angle is given by cos θ* = *r*_0_/*r*. The existence of a traveling contact region is the key to explain how local strip sliding can drive motility.

How the crawling speed *U* in a confined Euglenid is related to geometry and internal mechanics is an interesting and non-trivial problem, but it can be illustrated in two limiting cases. In the first it is assumed that friction between the pellicle and the channel wall is much larger than the hydraulic resistance of the surrounding fluid. In this case, the contact region, of length *l*_*c*_, is assumed to be instantaneously at rest with respect to the channel walls. As a result, the time, *δt*, needed for the contact pulse to travel a distance *l*_*c*_ can be determined from the constraint of fixed pellicle area, leading to *δt* = *l*_*c*_ *r*/(*r*_0_ *c*) = *l*_*c*_/(*c* cos θ*). The distance that the cell’s left edge travels in this time is − *l*_*c*_ + *cδt*, and therefore the cell speed is *U* = *c*(1 − cos θ*). In this limit, channel fluid is pushed in the direction *opposite* to migration, in contrast with cortical-flow based movement in channels Bergert et al. (2015). This is because of fluid pumping created by rearward, peristaltic surface pulses. In the opposite limit, of large hydraulic resistance and essentially immobile channel fluid, an argument based on volume conservation can be made to show that *U* = *c*(1 − cos^2^ θ*). This is a larger migration speed than before, and shows, interestingly, that the roles of cell-channel friction and hydraulics are interchanged compared with cortical actin flow-based movement: friction hinders, while hydraulic resistance enables, cell migration. In between the two limits, the cell migrates using a superposition of both mechanisms.

*Euglena* motility exemplifies how single cells and single-celled organisms are not restricted to a single mode of migration, a recurring motif in cell motility. By subsuming the incompletely understood mechanochemistry of metaboly into a single parameter, *c*, and using this as a starting point, the study by Noselli et al. (2019) nicely illustrates the use of phenomenological models in interpreting complex biological observation. Migration mode switching in Euglenids is, furthermore, a remarkable example of a self-organized, biological phenomenon: using the same small-scale elements, i.e., microtubules, but applying different rules of interaction can lead to strikingly different macroscopic behaviors.

### 3.2 Amoeboid swimming

Up to now, the models that I have presented benefit from the simplicity of channel-based migration, which strongly constrains the allowed cell shapes. Amoeboid migration, as defined by the absence of specific adhesion complexes in generating propulsion force, is more general, and as the example of *Euglena* showed, can be driven by shape change. This occurs, for example, for neutrophils, *Dictyostelium* amoeba, and lymphocytes, which can “swim” in an unconfined fluid in response to a chemoattractant Barry and Bretscher (2010); Aoun et al. (2020). These observations have motivated a number of model approaches, both analytical and computational Farutin et al. (2013); Campbell and Bagchi (2017); Wang and Othmer (2016); Wu et al. (2015). Here, I will detail the model by Farutin et al. (2013) because it illustrates clearly the physics of self-deforming amoeboid migration.

As has been well documented, microscale swimming is quite different than at larger scales Elgeti et al. (2015), where inertia dominates and gliding can occur. More specifically, migrating cells in a fluid generate essentially zero Reynolds’ number (Re) flow. As a generous estimate, Re ≡ *ρRU*/*η* ≈ 10^–5^, taking *ρ* = 10^3^ kg/m^3^ for the density of the cell’s fluid surroundings, *η* = 10^–3^ Pa.s as its viscosity, *U* = 5 μm/min as the cell speed, and *R* = 100 μm as its typical size. In this realm, and in accordance with Purcell’s “scallop” theorem Purcell (1977), directed motion of an amoeboid cell requires non-reciprocal deformation of the cell surface, necessarily involving more than one degree of freedom. An alternating series of expansions and contractions of a spherical cell along mutually perpendicular axes, for instance, cannot cause directional motion. Inspired by the swimming capabilities of *Dictyostelium*, neutrophils, and protists of the Euglenid family, Farutin et al. (2013) proposed a minimal model of a surface deformation-driven swimmer in a Stokesian (i.e., zero Reynolds’ number) fluid. They modeled the swimmer as a quasi-spherical vesicle of fixed volume *V* = 4*πR* ^3^/3 (defining an effective radius *R*) and bound by an inextensible membrane with area *A* = 4*πR* ^2^(1 + *Γ*), with the relative excess area *Γ* = 0 in the case of a perfect sphere. The membrane condition acts as a global constraint on deformation and acts in a way that resembles the inhibitory action of membrane tension on actin-based protrusions Sitarska and Diz-Muñoz (2020).

In the model of Farutin et al. (2013), the total membrane force acting on an element of the quasi-spherical surface, at a position parameterized by the polar angle *θ* and at time *t*, can be broken up into an active, driving term and a passive, tension term: **F** = *F*_a_(*θ*, *t*) **n** + **F**_p_(*θ*, *t*). They assume that the driving force acts solely along the local surface normal, **n**, implying that the mode of transfer of momentum to the cell’s surroundings is not at all like the examples considered up to now. The tension force **F**_p_ assures membrane inextensibility and has both a normal component proportional to local membrane curvature (reflecting Laplace’s law) and a tangential component that relaxes away any local membrane stretching. To mimic what they call “amoeboid swimming”, they assume, without detailing a specific mechanism, that biochemical regulation dictates some functional form for *F*_a_(*θ*, *t*), which is periodic in time to reflect that motion consists of repetitions of a given stroke cycle. The cyclic **F**(*θ*, *t*) in turn generates a cyclic surface displacement denoted as *u*(*θ*, *t*). The key question is to understand how *u*(*θ*, *t*) leads to swimming motion.

By considering only axisymmetric shape modes of a quasi-sphere, Farutin et al. (2013) were able to reduce a complicated problem to a system of three coupled differential equations for three time dependent quantities. This was done by first expressing *F*_a_ and *u* in terms of “normal modes”: for example, $u\left(\theta ,t\right)=\sum_{l=2}^{\infty }u_{l}\left(t\right)P_{l}\left(\cos\theta \right)$ , with *u*_*l*_(*t*) the mode amplitude and *P*_*l*_(cos *θ*) a Legendre polynomial; this is analogous to a Fourier superposition, but on a sphere. Here, *l*/*R* can be interpreted as the “wavevector” associated with mode *l*. The expansion is simplified by only considering the *l* = 2 and *l* = 3 modes, providing two degrees of freedom that should enable directed swimming; see Figure 2B. (The *l* = 1 mode is excluded since *P*_1_ = cos(*θ*) corresponds to a small, rigid translation of the sphere, and cannot contribute to swimming.) The three variables are therefore the amplitudes *u*_2_(*t*) and *u*_3_(*t*) and the cell velocity, *U*. The amplitudes are linked by the constraint of fixed membrane area, and their dynamics are determined by balancing the normal component of **F** with the reaction force exerted by the surrounding fluid. Finally, *U* is determined by a purely geometric equation relating it to the time derivative of the shape amplitudes.

In spite of its relative simplicity, the quasi-spherical swimmer model presents some surprising results. First, Farutin et al. (2013) show that the vesicle can move even though *u*_2_(*t*) and *u*_3_(*t*) are coupled by the area constraint. This seems to be at odds with the scallop theorem, forbidding migration involving a single degree of freedom. The paradox is resolved by noting that for any value of *u*_2_, the constraint admits two possible values of *u*_3_. In fact, a swim cycle can be represented as a closed ellipse in the *u*_2_ − *u*_3_ plane, and the vesicle displacement along the symmetry axis (*x*) during one cycle, Δ*x*, is related to the ellipse’s area. Up to a numerical coefficient, Δ*x* can be expressed as the product of *R Γ*, a purely geometric quantity. Surprisingly, this result is independent of the active force distribution. Even if the vesicle is not a quasi-sphere, i.e., *Γ* is not close to zero, Δ*x* remains roughly proportional to *Γ*, and is intuitively understood since greater *Γ* implies larger surface deformations, and hence greater deformation-induced outer flow, and therefore larger momentum transfer.

The authors then ask how, within the framework of the quasi-spherical model, the swimming speed is optimized. This question is important since it relates to migration efficiency and hence to energy consumption Yang et al. (2021). It is also of practical interest in this model because a general active force distribution *F*_a_(*θ*, *t*), and hence a general surface displacement *u*(*θ*, *t*), will involve higher harmonics than just *l* = 2 and *l* = 3. What rule should be applied to determine the force amplitudes? This question is answered by looking for the distribution of the *F*_*l*_(*t*)’s that will lead to the largest center of mass displacement per cycle. Interestingly, up to a numerical coefficient, this optimal displacement is again related to the geometric quantity *R Γ*. Therefore, cell geometry is an important predictor of migratory ability of amoeboid swimmers.

Can amoeboid swimmers be classified as pushers or pullers, like other moving cells? Farutin et al. (2013) argue that this binary distinction does not apply here. Indeed, because the cell shape is not stationary there are puller *and* pusher phases during a swim cycle. During a stroke in which the cell is elongating along, and narrowing perpendicularly, to the *x*-axis, fluid is pushed away along ± *x* and pulled in towards the cell in the plane perpendicular to *x*. The opposite fluid motion occurs when the cell is contracting along *x* and expanding in the perpendicular direction. Therefore, in contrast with shape-invariant migrating bacteria Elgeti et al. (2015), crawling cells Mogilner and Keren (2009), and amoeboidally migrating Walker carcinoma cells Bergert et al. (2015), amoeboid swimmers such as neutrophils and protists exhibit a hybrid puller/pusher behavior. One might speculate that this flexibility allows a self-deforming cell to adjust its gait depending on the nature of the surrounding fluid or matrix.

## 4 Migration and symmetry breaking

Cell migration is contingent upon a prior symmetry breaking event that polarizes distributions of motility-related proteins and signaling pathways. This is a general phenomenon, and applies to the different types of polarized surface movements considered in Sections 2 and 3. Polarity can manifest in surprisingly diverse ways. For example, a front-back asymmetric distribution of membrane water and ion channels results in osmotically-driven, cytoskeleton activity-independent cell migration in confined channels Stroka et al. (2014). Yet, in all of these examples, the motile mechanism is treated as a distinct module from the upstream mechanisms that polarize the cell. Yet, observations that immobile, non-polarized cells can spontaneously polarize and move suggest that motility and cell symmetry breaking are linked Verkhovsky et al. (1999); Yam et al. (2007); Ruprecht et al. (2015). Reconstituted systems have corroborated this idea: for instance, actin shells growing on nucleator-coated beads eventually rupture under elastic stress; the ensuing polarized polymerization results in bead motility van der Gucht et al. (2005). These findings are in line with now well-established proof that feedback between mechanics and chemistry exists in cells and tissues, notably in the actomyosin cytoskeleton Hannezo and Heisenberg (2019). For example, myosin-generated cortical actin flows can transport regulatory molecules thereby modifying the flow. Also, the nonlinear kinetics of actin polymerization regulators can give rise to non-trivial interplay between chemistry and mechanics.

Because adhesion complexes are absent in amoeboidally migrating cells, cortical flows can be long-ranged and therefore effective in mediating mechanochemical feedback, in addition to transferring momentum between cell and environment. This suggests that cortical flow can play a dual role in transporting molecules to establish front-back cell asymmetry *and* driving migration. Here, I will first review a modeling approach motivated by observations of quasi-spherical cells that self-polarize and migrate amoeboidally using cortical flow, all the while keeping a fixed shape Poincloux et al. (2011); Ruprecht et al. (2015); O’Neill et al. (2018); Aoun et al. (2020). The simplicity of this geometry helps to identify clearly the physics of self-organized motility. It also provides a first clue into how deterministic models can give rise to irregular cell trajectories Lavi et al. (2016); Stankevicins et al. (2020); Ecker and Kruse (2021). In the second part of this section, I will briefly outline a possible mechanism where cell surface flows, regulator transport, and shape change lead to symmetry breaking and could enable a novel amoeboid mechanism based on peristaltic wave propagation.

### 4.1 “Squirming” of quasi-spherical cells

Cells that are suspended in a viscous fluid, keep a nearly spherical shape, and migrate using tangential cortical flows are reminiscent of squirmers, a generic model of self-propelled, spherical particles Lighthill (1952); Blake (1971). In this model, the surface flow, *v*_*s*_, is prescribed (for example, flow generated by surface cilia beating), and hence the particle velocity is uniquely determined from the solution of Stokes’ equation in the outside fluid. For cells *v*_*s*_ is unknown and therefore the relation between migration and surface flow is not as clear. To get around this difficulty a number of models have recognized that the physics of cortical flow-based migration is intertwined with the mechanisms leading to cortical polarization in the first place. Models based on active gel theory Jülicher et al. (2007); Prost et al. (2015) have shown that above a threshold contractility, an initially uniform and quiescent spherical cortex of radius *R* will spontaneously polarize and flow axisymmetrically Hawkins et al. (2011); Ruprecht et al. (2015); Callan-Jones et al. (2016); Farutin et al. (2019); Mietke et al. (2019a). In these models, a steady-state flow is maintained by contractility and actin turnover. With increasing contractility, generic modeling has shown that instability of the cortex first occurs for the lowest non-trivial Legendre mode, *l* = 1, representing a polar symmetry breaking. This occurs because viscous stresses associated with flow and diffusive flux associated with regulator concentration changes oppose cortical polarization, and these penalize modes with higher wavevectors.

These models suggest that once polar symmetry is broken, cortical flow from one pole to the other will, by exchanging momentum with the surroundings, lead to net motion. But what kind of motion ensues as the initial instability evolves and nonlinearities become felt? Is it persistent along one direction? Or perhaps oscillatory, or even more erratic? This is an important question, as it relates to the nature of amoeboid trajectories and migration efficiency. For now, we can get some insight by considering how different stabilizing effects interact near the contractility threshold for instability of the initial, homogeneous cortex. To do so, I will outline a simplified one-dimensional version of the stability analysis of Farutin et al. (2019), which neglects the cell curvature but captures the main qualitative features. This approach is inspired by the theory of mechanochemical patterns in active fluids Oster and Odell (1984); Bois et al. (2011) that has motivated one-dimensional self-polarization/migration models Callan-Jones and Voituriez (2013); Recho et al. (2013); Kimpton et al. (2013); Drozdowski et al. (2021).

In this description, the actin concentration, *a*(*x*, *t*) and bound myosin concentration, *m*(*x*, *t*), evolve according to two conservation equations: *∂*_*t*_ *a* + *∂*_*x*_(*av*) = −*k*(*a* − *a*_0_) and $\partial_{t}m+\partial_{x}\left(mv\right)=D\partial_{x}^{2}m$ . Here, *v*(*x*, *t*) is the velocity field along the *x* axis that advects actin and bound myosin; *k* is the actin turnover rate and *a*_0_ is the homeostatic, uniform filament concentration; and *D* is the thermal diffusivity of actin-bound motors. A third equation comes from force balance along *x*, expressed as *∂*_*x*_ *σ* = 0, where the stress, for a 1D active fluid, is *σ* = *η∂*_*x*_ *v* + *ζf*(*m*) − *βa*. In this equation *η* is the cortex viscosity; *ζ* > 0 is the contractility coefficient, and *f*(*m*) is some monotonically increasing function of myosin concentration; and finally *β* > 0 is a compression modulus of the actin network, acting like a one dimensional pressure. For simplicity, we assume *f*(*m*) = *m* and that friction with a substrate, which would appear as *αv* on the right-hand side can be neglected. Therefore, this model is useful to understand the nature of self-polarization and flow that, with a momentum absorbing substrate or fluid present, would drive migration.

**FIGURE 3:**
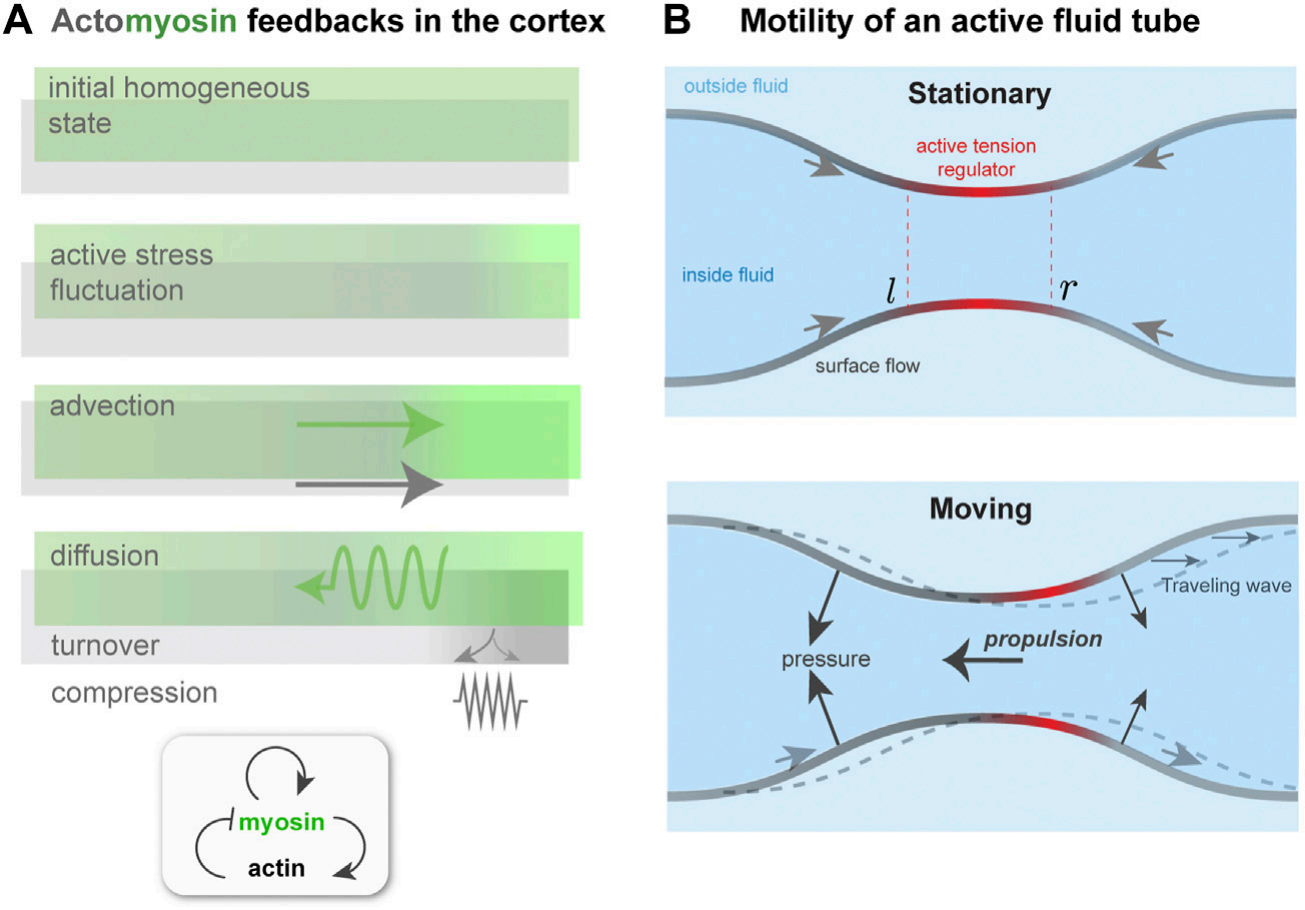
Contractility regulation in the cortex, flows, and symmetry breaking. **(A)**. Mechanochemical feedback in a simplified one dimensional actomyosin cortex. A localized increase in bound myosin concentration (green) creates higher contractile stress than elsewhere. The resulting force advects myosin, tending to reinforce the initial increase, which is counteracted by diffusion. Co-advection of actin (gray) has an inhibitory effect, because of opposing compressive stress and turnover, which penalizes density changes away from the initial, homeostatic state. In the inhomogeneous patterned state, steady-state state flows, in the presence of a momentum absorbing substrate, can give rise to migration. **(B)** Proposed migration mechanism based on theory of self-organized active tube patterning Mietke et al. (2019b). A contractility-based instability on the tube surface leads to an inhomogeneous active stress regulator profile, as in panel **(A)**. This concentrates the active stress and pinches the tube. A situation where the regulator peak is centered on the pinch (Stationary) may be unstable (see text), giving rise to a broken symmetry state. This state is characterized by peristaltic surface waves; in the presence of an outer fluid, directed motion would occur.

To summarize so far: *ζ* > 0 is the driving parameter that, if sufficiently high, causes instability, whereas *k*, *D*, *η*, and *β* are stabilizing parameters. In a suitably non-dimensionalized form, only three of the four will appear independently. For the stability analysis around the initial, homogeneous, non-flowing state (*a* = *a*_0_, *m* = *m*_0_, *v* = 0), let *a* = *a*_0_ + *δa*, *m* = *m*_0_ + *δm*, *σ* = *σ*_0_ + *δσ* and linearize the two advective terms, assuming *v* is small. Next, the two dynamical equations are Fourier-transformed with respect to position *x*, i.e., taking *δa*, *δm*, *v* and *δσ* to be ∝ *e* ^*iqx*^ and redefining *a*, *m*, *v*, and *σ* as the corresponding mode amplitudes. The velocity *v* and stress *σ* can be eliminated and the dynamical problem can be expressed as *∂*_*t*_ *a* = −(*k* + *β*)*a* + *ζm* and *∂*_*t*_ *m* = −*β a* + (*ζ* − *Dq* ^2^)*m*. Note that *η*, *a*_0_, and *m*_0_ have been set to one for readability; this does not affect the final results. These coupled equations resemble a model of reaction-diffusion in which *m* could be thought of as an activator and *a* as an inhibitor; see Figure 3A.

The above equations can be combined to give $\partial_{t}^{2}m+\mu \partial_{t}m+\omega_{0}^{2}m=0$ . This is analogous to the equation of motion for the position *m*(*t*) of a damped harmonic oscillator, where we identify *μ* ≡ *k* + *β* + *Dq* ^2^ − *ζ* as the “friction coefficient” (which may be negative) and $\omega_{0}\equiv {\left[Dq^{2}\left(k+\beta \right)-k\zeta \right]}^{1/2}$ as the “natural frequency” (which may be imaginary). Assuming that small perturbations vary as *m* ∝ *e* ^*λt*^, their growth rate is given by $\lambda_{\pm }=-\frac{\mu }{2}\pm \frac{1}{2}\sqrt{\mu^{2}-4\omega_{0}^{2}}$ . Instability occurs if the real part of *λ*_+_ goes from negative to positive; beyond the linear regime the instability will evolve into some kind of pattern. Surprisingly, this model predicts two qualitatively different kinds. A first one can occur if $\mu^{2}>4\omega_{0}^{2}$ , i.e., the oscillator is overdamped, and the *λ*_±_ are real. A steady-state bifurcation then takes place if *ω*_0_ goes from positive to imaginary, a condition that yields the critical contractility *ζ*_*c*_/*Dq* ^2^ = 1 + *β*/*k*. To go back to the original problem on a sphere, one identifies *q* ∼ *l*/*R*, and therefore with increasing contractility the polar mode, *l* = 1, will be the first to become unstable. Above *ζ*_*c*_ the instability can be expected to develop into a polarized steady-state associated with persistent migration. A second kind of pattern can occur if $\mu^{2}<4\omega_{0}^{2}$ , corresponding to an underdamped oscillator. A so-called Hopf bifurcation takes place when *μ* goes from positive to negative, yielding a different critical value, *ζ*_*c*_/*Dq* ^2^ = 1 + (*k* + *β*)/*Dq* ^2^. Above this contractility threshold the instability is expected to develop into a non-steady, oscillating pattern; in other words, a standing wave and non-persistent migration.

How does one know what type of pattern, and hence motility, will occur in this model? A Hopf bifurcation happens if *ω*_0_ is real at threshold, or in other words if *Dq* ^2^ *β* > *k*(*k* + *β*). Thus, competition between the different stabilizing terms in the problem—diffusion, cortical compressibility, and turnover—will affect the nature of the pattern and hence the type of cell motion. This is the main point of this model: antagonism between a small number of driving and stabilizing effects gives rise to subtle emergent migration behavior. Yet, much has been neglected here, and one can rightly ask, how much predictive value does this type of simple model have for amoeboid migration? Answering this would require that the model parameters be experimentally measurable, along with observation of different migration phenotypes. The model emphasizes the patterning effect of advection by cortical flow, and as such only considers one part of a large mechanochemical parameter space; models that explore differents parts of it will give rise to different predictions. For example, local shape changes could directly or indirectly alter motility Bodor et al. (2020). Indeed, competition for actomyosin by endocytic machinery has been shown to lead to oscillatory trajectories Lavi et al. (2016). Other types of self-organized migration behavior have been predicted by models in which advection-type nonlinearity is absent Doubrovinski and Kruse (2011); Ziebert et al. (2012). For example, theoretical studies have explored the impact of the cooperative kinetics of actin assembly promoters, such as Rho-GTPases, on cell polarity patterns, migration, and trajectories Camley et al. (2013), Camley et al. (2017); Ecker and Kruse (2021); Stankevicins et al. (2020). Finally, coupling cell shape to migration machinery constitutes a thinly explored region of parameter space. This has been touched on in modeling based on actin regulator nonlinearities Camley et al. (2013); Ecker and Kruse (2021), but not yet in the more prevalent situation of cortical actin-based amoeboid migration. A hypothetical scenario illustrating this coupling is considered in the next subsection.

### 4.2 Peristaltic wave-based migration

As the cortex of a model spherical cell polarizes, with actomyosin enriched at one pole and flows directed between poles, the cortical tension becomes inhomogeneous. As a result, Laplace’s law tells us that shape change occurs, the surface curvature varies with position, and the cell is no longer spherical Bun et al. (2014); Callan-Jones et al. (2016); Wu et al. (2018); Le Goff et al. (2020). This, then, raises the question of whether the deformation of the cortical layer feeds back in any way on the cortical flow, and if so, is the mechanism of migration altered.

To attempt an answer to this, I will sketch how the interplay between surface flows and shape change can give rise to a type of migration, based on peristaltic surface waves, that is qualitatively different from those considered earlier. This idea is motivated by recent work on mechanochemical patterning of active fluid surfaces Mietke et al. (2019b). As done in Mietke et al. (2019b), I consider a tubular surface composed of an active fluid, which could represent the actomyosin cortex (Figure 3B). The active, contractile tension in the surface is controlled by a regulator species that diffuses and is advected by surface flow. For simplicity, I identify this with the bound myosin concentration, *m*, as done in the previous subsection. A homogeneous tube of radius *r*_0_, uniform *m* and no surface flows will, above a threshold contractility *ζ*, destabilize and form concentration bands of regulator and steady-state surface flows, just like for one-dimensional mechanochemical patterns Bois et al. (2011).

A key difference here is that the inhomogeneous active tension will also deform the surface Mietke et al. (2019b); in particular, a regulator band acts like a contractile ring, squeezing the tube. The extent of squeezing is limited by the bending rigidity, *κ*, of the surface; if *κ* = 0, above threshold the tube will pinch to zero radius where the regulator is most concentrated Mietke et al. (2019b). However, for finite *κ*, the tube radius remains finite at long times, and furthermore the position of regulator bands and the flow field undergo a symmetry breaking. The origin of this can be understood with a qualitative argument. With reference to Figure 3B, a slight left shift of the regulator band, and hence of the active tension profile, with respect to the surface leads to squeezing near *l* and expansion near *r*. This, in turn, further concentrates the regulator near *l* and dilutes it near *r*. Therefore, both the surface deformation and the regulator profile get shifted to the left; there is no restoring force that keeps the regulator maxima in phase with the surface radius minima. Finally, the shift in regulator *m* also affects the force balance in the plane of the surface, and causes a shift in the flow profile *v* with respect to the surface. As a result, the symmetry of the surface is broken; in a reference frame in which the period-averaged surface flow vanishes, one would see co-moving waves of regulator concentration and of surface shape Mietke et al. (2019b).

How does this symmetry breaking relate to amoeboid migration? The directed surface flow and peristaltic shape waves could both generate propulsion force. If the active tube were confined in a fluid-filled channel, flows on the surface where the tube radius is largest would exchange momentum with the channel wall, by lubrication forces, for instance, and hence create propulsion. Alternatively, if the tube is suspended in a fluid and peristaltic waves travel with respect to it, then the pressure distribution on the tube will change: In regions where the tube is expanding the outer pressure increases, and where the tube is contracting the pressure decreases. This gives rise to a propulsion force, similar to the case of topography-based motility in Section 2.2.

The idea of using surface waves to propel a cell was actually proposed long ago by Abercrombie et al. (1970), though the engine of motility was not known. Here, I have proposed that, similar to squirming cells, the same physics governs the symmetry breaking of the surface *and* determines the source of propulsion force. Peristaltic wave-based migration is reminiscent of amoeboid migration of slime mold, which is dependent on actomyosin contractility Alim (2018). In that case, though, waves are thought to be coordinated by bulk, as opposed to surface, contractility and cytoplasmic flows Lewis et al. (2015). Perhaps closer to the mechanism proposed here is recent observation of amoeboid migration of fat body cells of *Drosophila* Franz et al. (2018). There, movement of a contractile ring with respect to the cell appears to coordinate migration, though how propulsion force is generated is not fully known.

## 5 Concluding remarks

This paper has surveyed a range of surface movements and self-organized behavior constituting models describing cell migration in the absence of adhesion complexes. These models are expressed in terms of flow velocity fields, surface displacement, and concentrations of regulators. The same physical quantities also describe cell functions and a large range of developmental movements not associated with cell migration: for example, contractile ring formation prior to cytokinesis and mechanochemical patterning in the developing organism. As such, there is general interest in forming a modeling framework for macroscopic phenomena in cell and tissue biology, in which amoeboid migration is one example.

In formulating internally consistent theoretical models, for the sake of simplicity certain variables are neglected, even though their possible impact is often difficult to assess. One example is to what extent organized actin architectures, for instance, isotropy *versus* anisotropy of filament ordering, is an important variable in migration models. Long-range alignment of actin filaments exists in the lamellipodium of mesenchymally crawling cells Verkhovsky et al. (2003); cortical actin filament alignment in cortical-flow driven amoeboidally migrating cells also been documented Ruprecht et al. (2015). While meso-level filament alignment has been included in some migration models, specifically in cases where the flow field is not explicitly considered Ziebert et al. (2012); Dreher et al. (2014); Ecker and Kruse (2021), little is known as to whether its inclusion qualitatively affects the basic mechanisms of adhesion-free, amoeboid migration. Some positive evidence in this direction is suggested by abstract migration models. For instance, a model of a drop of active fluid on a substrate has shown that spatially varying orientational order can mediate self-propulsion in the absence of local traction forces Loisy et al. (2019). It will be interesting to pursue this area, in particular in joining theory with experiments that can probe cortical organization together with migration.

Membrane tension is another variable whose influence on amoeboid migration is not well understood. For substrate-borne, crawling cells, in contrast, it is known that membrane tension has a regulatory role in actin protrusions, including the lamellipodium Diz-Muñoz et al. (2013); Lieber et al. (2013). However, because actin polymerization interacts with the membrane in a fundamentally different way in the two migration modes, the role of tension is expected to be different. A recent study of the motility of macrophages suspended in fluid suggested that cortex-linked membrane flows create viscous stresses in the fluid thereby providing propulsion O’Neill et al. (2018). How membrane turnover enables these flows is not known, however. Furthermore, whether membrane flow and the concomitant tension changes affect internal activity, for instance *via* contractility signaling, has not been explored.

Even relatively simple models (Section 4) of cytoskeletal self-organization predict non-trivial cell polarization patterns and consequently different gaits and trajectory types. Including internal noise in a minimal, one-dimensional mechanochemical model of myosin advection-based symmetry breaking results in persistent or intermittent motion, depending on the strength of advection coupling Maiuri et al. (2015). By accounting for other dynamical features, for instance, feedbacks associated with actin polymerization at the membrane significantly enlarges the mechanochemical parameter space. As a result, even in purely deterministic settings erratic, random walk-like trajectories can be predicted Stankevicins et al. (2020); Ecker and Kruse (2021). Given the importance of a migrating cell’s search efficiency in living systems, understanding the connections between internal activity and long-time migration dynamics is a fertile area of research.

A number of types of motile cells and single-celled organisms, when found in surroundings where their “default” mode—for instance, adhesion complex- or flagella-based—is not viable, have evolved alternative migration modes involving different physical principles. But how does the cell “know” which mode to choose, given its circumstances? How does the cell interpret cues from its environment, and is there a simple metric that the cell evaluates to make its decision? How much do considerations of metabolic efficiency play a role Yang et al. (2021)? To what extent physics can provide answers is not known, but these are still useful questions to contemplate in order to interrogate more generally how out-of-equilibrium systems “decide” between multiple states. Migration models consisting of droplets of active matter might be a useful, though reductionist, means of tackling this problem Tjhung et al. (2012). Recent modeling has treated migration as an inverse problem, in that it asks what is the optimal functional form of the contractile stress in order for the drop to get from one point to another Recho et al. (2014); Shankar et al. (2021). These models proceed by minimizing some cost function related to mechanical work done and internal resources used by the cell, and find the active stress that generates motion. Yet, these models pre-suppose a particular migration mechanism, and the mechanism for upstream choice of which mode to choose from is left open. Though far removed from the complexity of a whole cell, some inspiration might come from thermodynamic control theory applied to networks of out-of-equilibrium filaments, such as the cytoskeleton Lamtyugina et al. (2021). Based on this work, it has been suggested that energy resources and external cues could effectively modify cytoskeletal material properties and drive transitions between many-filament structures Lamtyugina et al. (2021). It will be interesting to apply this approach to active droplets in which different migration modes can be associated with dynamic structural transitions of the material.
